# Data Modifications in Blockchain Architecture for Big-Data Processing

**DOI:** 10.3390/s23218762

**Published:** 2023-10-27

**Authors:** Khikmatullo Tulkinbekov, Deok-Hwan Kim

**Affiliations:** Department of Electrical and Computer Engineering, Inha University, Incheon 22212, Republic of Korea; 22202364@inha.edu

**Keywords:** blockchain, IoT, big data, data modifications, selective deletion, edge computing

## Abstract

Due to the immutability of blockchain, the integration with big-data systems creates limitations on redundancy, scalability, cost, and latency. Additionally, large amounts of invaluable data result in the waste of energy and storage resources. As a result, the demand for data deletion possibilities in blockchain has risen over the last decade. Although several prior studies have introduced methods to address data modification features in blockchain, most of the proposed systems need shorter deletion delays and security requirements. This study proposes a novel blockchain architecture called Unlichain that provides data-modification features within public blockchain architecture. To achieve this goal, Unlichain employed a new indexing technique that defines the deletion time for predefined lifetime data. The indexing technique also enables the deletion possibility for unknown lifetime data. Unlichain employs a new metadata verification consensus among full and meta nodes to avoid delays and extra storage usage. Moreover, Unlichain motivates network nodes to include more transactions in a new block, which motivates nodes to scan for expired data during block mining. The evaluations proved that Unlichain architecture successfully enables instant data deletion while the existing solutions suffer from block dependency issues. Additionally, storage usage is reduced by up to 10%.

## 1. Introduction

In recent years, blockchain technology has emerged in several fields. Distributed and secure peer-to-peer (P2P) networks were first employed in the financial world, leading to the emergence of new cryptocurrencies and nonfungible token (NFT) exchanges, such as Bitcoin [1] and Ethereum [2]. These dramatic advancements in modern financial systems have attracted academic interest in the integration of blockchain into numerous other fields [3,4,5], including edge computing [6,7,8]. Edge computing enables a decentralized approach to Internet of Things (IoT) data processing to address the centralization limitations of cloud-based data centers. The edge nodes are located geographically close to the user plane, which allows localized data handling. Edge computing employs distributed edge nodes, making it a likely candidate for implementing blockchain protocols. Recent architectures, such as Recordchain [9] and Groupchain [10], have demonstrated the benefits of blockchain integration in edge-computing environments.

However, the data processing requirements of these two systems present challenges owing to technological mismatch. Edge-computing nodes typically handle IoT devices that generate big data through frequent data updates and deletion operations. However, a traditional blockchain requires all nodes to share the same database for reliability and immutability, thereby preventing data alteration upon insertion into the blockchain. Because all nodes share a single copy of blockchain data, the network can easily reject any malicious modification. This simple rule has been implemented in cryptocurrencies since 2009, enabling a secure money-exchange protocol without government interference. In 2014, a network called Ethereum [2] offered expanded blockchain capabilities with the introduction of a smart contract, an executable source code deployed in a blockchain, with execution on an Ethereum virtual machine (EVM). As a smart contract can include logic, it allows data modification. However, limitations in size and cost render smart contracts inapplicable to big-data processing.

Recently, academic attempts have been made to enable data modification in blockchain-based IoT environments. Although many state-of-the-art methods have been developed [11,12,13,14], no complete approach handles both big data and instant modification operations. Furthermore, most existing approaches fail to retain the security advantages of blockchains because the longest-chain rule is broken by deleting existing blocks. Most of these approaches require data with predefined lifetimes to enable deletions in the blockchain architecture.

Motivated from the limitations mentioned above, this paper proposes a new architecture called Unlichain (short for Unlimited Blockchain), which inherits all blockchain features while enabling predefined and on-demand data-modification operations. Similar to the existing state-of-the-art blockchains, Unlichain employs full and light nodes for scalability. In addition, Unlichain extends light-node capabilities with metadata-based transaction verifications to accelerate the confirmation speed. Unlike other existing approaches, Unlichain focuses on integrity, even for modified and deleted records, and employs a new block/transaction-indexing method with the possibility of hash and status referencing. Based on these new indexing methods, Unlichain achieves immediate deletion over predefined lifetime records. Moreover, on-demand modifications can be performed using delete/update API. Consequently, Unlichain exhibited advantages in storage utilization while retaining the longest-chain rule, proving its effectiveness in terms of security. Within this scope, this study makes four main contributions:

- Delay-free metadata-based verification of new transactions to achieve high-throughput;
- New block-indexing method to maintain blockchain reliability while enabling deletions;
- Automatic deletion of data with predefined lifetimes;
- Possibility of on-demand deletions through API for undefined lifetime transactions.

The remainder of this paper is organized as follows. Section 2 discusses the related work, and Section 3 discusses the background and motivations for our research. Section 4 discusses Unlichain architecture and its newly employed features, and Section 5 presents a detailed discussion of the data-modification features. Section 6 presents the evaluation of the proposed method and discusses the experimental results. Finally, Section 7 concludes the paper.

## 2. Related Work

Because the original purpose of blockchain was to create a distributed and immutable database with high emphasis on data security, data-deletion operations were initially considered unnecessary. As the number of application fields has increased, many researchers have begun to consider the use of blockchain in big-data-oriented systems. Although data-deletion techniques have not been explicitly discussed in most related studies, these approaches have focused on enabling new research topics. This section discusses these state-of-the-art projects, most of which are related to data modification in the blockchain.

This research originated from Ethereum [1], which was introduced as a breakthrough in blockchain technology with the ability to store executable source codes. This has motivated developers to digitalize physical and virtual assets and store them in secure blockchain networks for secure ownership. In addition to their ability to store and execute logic, smart contracts have also been extended to enable data modification. By calling the correct logic, developers can update and delete the existing data within a smart contract. Motivated by smart contracts, projects such as Binance Smart Chains [15], polygons [16], and Solana [17] have been developed. However, the underlying blockchains continue to extend most of the consensus from Bitcoin, in which all databases must be broadcast on the network. This incurs limitations in terms of the latency and storage costs. To avoid these issues, Ethereum limited the size of each smart contract to 24 KB. Furthermore, the publication of a smart contract incurs a fee that will be given to the nodes as a reward for securing the network. In other words, these requirements have motivated developers to avoid excessively large smart contracts, owing to increased costs. Although this is sufficient for digitalizing valuable assets and enabling businesses to use new types of cryptocurrencies, it is not affordable in edge-computing environments. Hyperledger Fabric [18], on the other hand, introduces the method called “data pruning” and “private data collections”, which allows the data to have an expiration time and can be deleted later. Even though it seems like a solution for data modification issues, Hyperledger is only available as a private or permissioned network where the deployment and maintenance costs are affordable by only big companies and enterprises. Additionally, due to unpredictable network constraints, the exact data pruning solutions cannot be applied in public networks. As an alternative, Sayeed et al. [19] proposed a trustworthy and privacy-preserving framework named TRUSTEE, integrating Hyperledger Fabric, IPFS, and the latest encryption techniques for all data operations. Nevertheless, the usage of permissioned networks makes it impractical in public environments. IPFS is also widely used to integrate different blockchains as data storage [20]. For example, de Brito Gonçalves et al. [21] proposed IoT data storage on IPFS with integration of Ethereum smart contracts. This approach can be promising for precious data management, but integrating smart contracts makes it not affordable in typical big-data systems.

Another deletion-oriented method was introduced by Yang et al. [22] who developed a blockchain-based deletion technique for cloud storage. The authors enhanced the cloud server honesty by employing blockchain network verification for data modification. However, this technique does not fully focus on deleting blockchain data, but on securing deletion operations using blockchain. Zhu and Kouhizadeh [23] employed blockchain because of its traceability features in supply-chain systems where redundant data deletions are common. Here, a blockchain is implemented to avoid unintended product deletions and recover deleted data using traceability features. Bosona et al. [24] and Li et al. [25] also worked on the traceability and access management solutions on supply chain using the blockchain technology. El Khanboubi et al. [26] employed a blockchain protocol to enable the smart deletion of duplicated data. In this method, deduplication is easily verified using blockchain features, and automatic deletions are enabled for duplicate data. Li et al. [27] introduced another state-of-the-art approach for data auditing in cloud computing using a blockchain protocol that automates data management. Ra et al. [28] propose a blockchain-based XOR global-state injection method for content modification. Moreover, Kim et al. [29] proposed an evaluation model to measure the immutability in different blockchain technologies. However, all the aforementioned methods focus on employing blockchain as an additional feature to enable or monitor data modification in cloud-based systems. Another state-of-the-art approach for secure data management was introduced by Xu et al. [30] with the implementation of a distributed redactable blockchain free of third-party participation. Guo et al. [31] also proposed transaction redaction features in a policy-hidden manner using blockchain. Lu [32] and Valadares et al. [33] compiled a survey discussing current issues and research gaps pertaining to the use of blockchain in big-data systems and their privacy features.

Although data deletion from blockchain has not been a central topic in most previous studies, the adaptation of blockchain to big-data systems has been a research subject for a long time. Huang et al. [34] proposed the BlockSense architecture, which is a fully distributed approach to mobile crowd-sensing techniques using a proof-of-data consensus. The authors achieved promising results in terms of data privacy and performance compared with the Ethereum network. Taloba et al. [35] proposed a hybrid platform for multimedia data processing designed for IoT–healthcare systems to manage patient-related data. Heo et al. [36] proposed a storage optimization technique with the help of employing blockchain for distributed caching. Zhaofeng et al. [37] introduced a trusted data management system for edge computing. Umoren et al. [38] proposed decentralized storage for user authentication in fog computing. Kwak et al. [39] proposed a blockchain-based solar energy trading platform mainly applicable for a smart city environment. On the other hand, Lian et al. [40] took a different approach to the meaning of big data by designing a secure and trusted system for storing large transactions generated by international trading. The IoTA Research Papers [41] employed tangle and coordinator nodes to maintain consensus in microtransactions. Xu et al. [42] introduced a trustless crowd-sensing technique for mobile edge using blockchain. Li et al. [43], MEVerse PTE Ltd. [44], and Chia Network [45] represent group-based consensus approaches for blockchain protocols to handle the higher throughput inherent in IoT data systems. Although these techniques do not focus solely on data deletion, the applied environment theoretically provides new research directions for general data modifications. In parallel to blockchain integration with big-data-related systems, its security is always becoming an emerging topic. In their survey paper, Yassine et al. [46] discuss the possible challenges and practical applications of blockchain in cybersecurity and data privacy. Ali et al. [47] also listed the cutting-edge secrets of cyberphysical systems in consortium blockchain. Hameed et al. [48] made even more narrowed discussions addressing the blockchain-based industrial applications, their perspectives, and possible security threads.

Summarizing, Table 1 compares the related literature regarding data modification requirements in big-data systems. Since not all related literature describes data modification, only the closely related works have been selected for comparison. As the table shows, as the basic blockchain architecture, Bitcoin only has security-related requirements regarding big-data handling. Ethereum, on the other hand, offers more complex membership rules with light and full nodes. Also, the smart contracts enable the possibility of data updates. LiTichain uses a permissioned network for faster block verification. It offers a state-of-the-art solution for block deletions, direct updates are not provided, and the longest-chain rule is broken due to deleted blocks. Hillman et al. employ data deletions and updates on public networks. But still, the longest-chain rule needs to be preserved. Hyperledger Fabric is a famous blockchain solution that already offers data pruning. Due to its permissioned nature, Hyperledger does not follow the longest-chain rule and does not affect its security. However, it still has limitations in terms of instant deletions. Even if the data expires on Hyperledger Fabric, its removal is delayed until the data pruning occurs. Also, Hyperledger Fabric uses a private database for an expirable database and only stores the hash in the blockchain, which means the corresponding hash is never deleted. Kuperberg et al. and Sayeed et al. employ their solution based on Hyperledger Fabric, and there are many similarities, except that Kuperberg et al. do not employ off-chain data storage. Kanboubi et al., on the other hand, take a different approach by using blockchain to control data deletions on the central cloud. For this purpose, the authors employ a private blockchain with authorized entity participation. With this help, they can achieve an advantage on instant deletions, but this idea does not directly employ deletion inside the blockchain structure. Guo et al. also use a permissioned blockchain and achieve similar achievements to others. IOTA is also included in comparisons despite not employing data deletions. However, IOTA stands as one of the popular public blockchains that provides the highest transaction confirmation at low cost. The table shows that most successful solutions are based on the permissioned or private blockchain. The reason is that consensus is more accessible in consortium blockchain when the block structure and rules are changed. However, the same rules do not apply to public networks. For this reason, Unlichain stands as the complete solution that offers data modifications in the public blockchain network.

## 3. Background and Motivations

Edge computing is a widespread concept with significant advantages in IoT environments [49,50,51]. Because they are applied in big-data-processing systems, data security and dynamic node management are key issues in establishing a reliable environment. By providing a distributed network, blockchain offers the easiest solution to these problems. These issues and their possible solutions are discussed in detail in related studies [9,10,52,53,54]. Particularly, Deepa et al. [52] writes about the increasing demand for blockchain technology in storing, sharing, and auditing big data and possible practical applications it may benefit. Because the objective of the present study was to enable data modification, the content presented herein relates to topics such as the maintenance of basic features in the presence of new features. When applied to an IoT environment, edge-computing stores unpredictable user data, where the majority is meaningful only for a short time. Considering the example of a connected-car environment, traffic data are valuable only if they can be updated regularly using the most recent information. Similarly, older data lose their value after the corresponding traffic problems are resolved and can be deleted to ensure storage efficiency. Because the time sensitivity inherent in IoT data requires appropriate data management, the blockchain network must fulfill these requirements for long-term support and avoid data duplication and excess storage costs.

### 3.1. Blockchain

**Figure 1:**
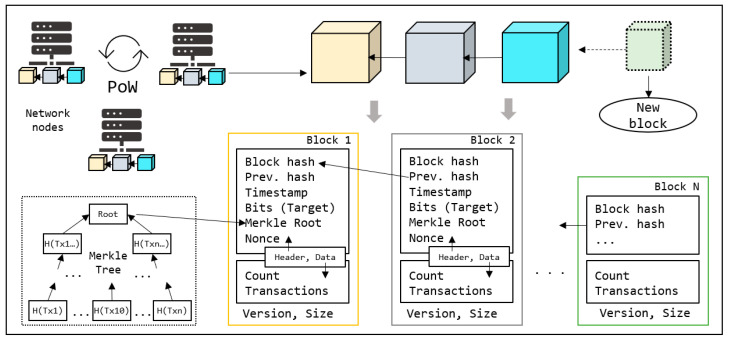
Blockchain overview.

Figure 1 presents a general overview of blockchain architecture. A blockchain is a database in which data is stored in linked blocks to form a chain. This database is shared in a P2P network that comprises several nodes with the same role. As shown in the figure, each block has header and data components alongside version and size information. The data component includes the list of transactions the block stores and header includes its metadata. Because this is a distributed network free of centralized authority, data management and processing are controlled by general agreement or consensus among nodes.

#### 3.1.1. Consensus

The main purpose of consensus is to enable data security and consistency in distribution, and to avoid byzantine nodes [55] that harm valuable data. According to this general agreement, the nodes decide whether to accept or reject each new block. A consensus is shared among all nodes, and any malicious activity or changes in general rules can be easily dismissed by the network. A traditional blockchain (e.g., Bitcoin) employs a proof-of-work (PoW) consensus, wherein all nodes are required to maintain a copy of the blockchain. Consensus starts by agreeing with the transaction list when added to a new block. In the case of Bitcoin, every node employs a data structure called Mempool, which stores all pending transactions to be saved in future blocks. Whenever a node receives a new transaction, it propagates to the network, allowing every node to add it to the Mempool. Each new block was created through a mining process. As the Mempool size is unbounded, the blockchain protocol sets a threshold for the block size. Subsequently, block mining is initiated by selecting the transactions. Due to size limitations, the nodes are expected to sort the pending transactions before selections. The selected transactions are stored in the data segment with the count, as shown in Figure 1. Based on these transactions, a unique Merkle root was generated and set with the other parameters in the header. After calculating the Merkle root, the hash of the final block was set to create a chain. This method assumes the content of a block and generates a unique hash for the subsequent block. This process challenges nodes in generating new nonce values, where H(nonce + data + metadata) satisfies a specified condition. The task of meeting this condition is referred to as difficulty and is updated dynamically by the network at certain intervals. Complexity increases when more nodes join a network. In Bitcoin, the block-mining difficulty is managed by the *Bits* field and usually set as a new block generation delay (approximately ten minutes). The first node that completes a job (work) propagates its results to the network for confirmation. The other nodes verify the calculations and respond to confirmation (proof). Upon receiving sufficient confirmation, the node appends a new block to the blockchain and receives rewards (coins) for its contribution. Thus, all nodes compete with each other for the right to create new blocks through mining. PoW is a time- and energy-consuming process that requires significant output from many nodes in the network. 

#### 3.1.2. Transaction Lifecycle

A transaction may have different forms and meanings based on the blockchain network’s purpose. In most cryptocurrencies like Bitcoin, the transaction refers to a crypto asset exchange between wallets. However, in smart contract-based blockchains like Ethereum, transactions may have more advanced forms like new smart contract creation or state changes. In all cases, a transaction refers to the data that needs to be saved in the distributed ledger. Compared to a centralized approach, validating transactions in a distributed network is a multistep process. The first challenge starts with the correct propagation to Mempool. Because Mempool is an in-memory data structure, nodes usually have different versions. When mining a block, the miner uses the available version, but it does not necessarily mean the latest Mempool. The second step would be a successful selection from Mempool. As explained in the previous section, the blocks are created in certain intervals and have size limitations. As a result, transactions are usually sorted by the transaction fee during the block mining. It may result in a long delay for the small transactions. To avoid delays, modern blockchains like the updated version of Ethereum offer a “*tipping technique*” in which transaction owners pay a tip for the miner to approve the transaction faster. The final step would be successfully winning the consensus. If another node with a different Mempool version wins the consensus, the transaction verification may be delayed until the next block creation. 

#### 3.1.3. Merkle Tree

As the blockchain is a distributed network, one of the biggest challenges would be avoiding multiple confirmations of the same transaction. A Merkle tree is employed as a part of block mining to achieve integrity. As shown in Figure 1, the Merkle tree uses the hashes of all selected transactions. One new hash is generated from each of the two hashes, and the process continues until only one hash is left in the root. The root is saved as a Merkle root in the block header. This structure allows for efficient and rapid verification of transactions: to prove the inclusion of a specific transaction within a block, one only needs to provide a path of hashes up to the Merkle root rather than the entire set of transactions. Furthermore, any alteration to a single transaction results in a change to the Merkle root, effectively highlighting any discrepancies in data. This mechanism greatly enhances the security and scalability of blockchains, making Merkle trees a foundational element in blockchain’s cryptographic toolkit. 

#### 3.1.4. Longest-Chain Rule

**Figure 2:**
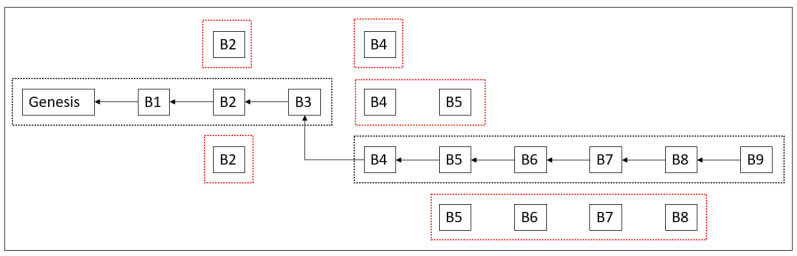
Longest-chain rule.

As mentioned previously, a traditional blockchain is designed for public networks, where integrity and security are the highest priorities. Owing to the nature of distributed computing, different versions of the blockchain may exist during block creation, as shown in Figure 2. The longest-chain rule has been proven to be the most effective approach in blockchain protocols to achieve universal reliability in public blockchains. According to this rule, the node that holds the longest chain achieves consensus and its blockchain is accepted by all nodes in the network. As shown in the figure, there is more than one possible candidate for a new block (B2, B4, or B5), shown as dotted squares in red color. A network usually struggles to choose which version of the chain to select and ignore. Therefore, blockchain nodes agree on the longest-chain rule, in which the nodes wait until one of the chains becomes longer with more blocks. In this scenario, all nodes constantly update their copies of the blockchain with a longer chain to keep their database up to date. Whenever one of the chains becomes longer as the figure shows in the order of dotted rectangles in black color, the chain can be accepted by the network and the other versions are ignored. If an attacker wants to modify existing blockchain data, they must create a longer chain with updated data and ensure acceptance by the network. However, the creation of new blocks requires heavy computation and network confirmation from at least 51% of nodes. Thus, an attacker cannot create a longer chain without a consensus. Any other trial is easily rejected by network nodes with the longest chain.

### 3.2. Data Modifications

As explained in the previous section, traditional blockchain consensus strictly prevents data modification after validating a block. This is the primary factor that maintains blockchain security and is the biggest problem in blockchain integration into big-data systems. Because the data are stored inside blocks and mutually linked using a hashing technique, there is no practical possibility of deleting or updating specific data inside these blocks. Instead, the simplest way to delete data from the blockchain is to delete the corresponding blocks without violating the chain protocol. Theoretically, there are two possible ways to enable block deletion without breaching the consensus: (1) deleting the last block prior to the next block confirmation and (2) deleting the first block by updating the genesis block location.

The last block deletion (LBD) method was proven to be practically effective by Pyoung and Baek, who implemented LiTichain [10] for finite-lifetime blocks. They created two graphs and sorted the blocks in descending order according to their lifetimes. To preserve the physical locations and sorting order, each block contains references to both the preceding and parent blocks. In this scenario, the block to be deleted first is situated last, thus allowing it to be deleted before the next block arrives. A deletion delay occurs if a subsequent block arrives before the last block is deleted. To minimize delays, LiTichain employs a k-height insertion method, in which blocks are not restricted to arrival-time ordering. Because the parent block-ordering chain is maintained, consensus can be reached by following the expiration time order. Although this approach represents a unique solution for implementing the LBD method, it is limited in terms of implementation because preprocessing is required to sort the data according to expiration time. Whenever a block is deleted, its entire data storage is lost; therefore, each block can only store data with a similar expiration time to maintain its integrity. Furthermore, block deletions are delayed if a child block has not yet expired and may consume excessive storage. Although LiTichain effectively enables block deletions, this end-to-end delay is not acceptable for edge computing. Furthermore, block deletion requires a predefined lifetime for each block, which in turn requires data with predefined lifetimes. Consequently, this method could not be applied to dynamic workloads.

The first block deletion (FBD) approach appears to be inefficient if changing hash references is required in the blockchain structure. Hillmann et al. introduced a possible implementation that enables selective deletion [11]. In this approach, a new summary block is created after a specific threshold, which does not store new data, but summarizes the previous blocks. In this scenario, the deleted data are skipped, and the new summary block references the genesis blocks directly. The references to the old blocks are then replaced with a new chain extended with summary blocks. This simple approach focuses on deleting the first block by logically forgetting the path, thereby enabling a new chain to store only the latest data. Although this technique may seem practical, easy, and effective, it also incurs a delay until the summary block is created, which has the same drawbacks as LiTichain in terms of edge-computing requirements. Moreover, both the methods focus on deleting entire blocks that may violate the longest-chain rule in a blockchain.

Considering the data-modification scenario, both the LBD and FBD approaches focus on deleting existing blocks from the blockchain, rendering the blockchain vulnerable to attacks. Therefore, the design of a new architecture with data-modification features requires careful consideration of the longest-chain rules for reliability and security.

### 3.3. Motivations

Because the traditional blockchain protocol prevents data modification after block verification, its integration into edge-computing faces issues related to big-data management. Although several existing state-of-the-art approaches enable data deletion, there is still a significant research gap in fulfilling all edge-computing requirements and security concerns. First, the deletion of all blocks from the chain violates the longest-chain rule, thereby losing the protocol reliability. However, selective deletion of data from within blocks is impossible because of complex hash-based calculations. Furthermore, the deletion of entire blocks requires a predefined data lifetime, which cannot be ensured by on-demand data deletion. Although several studies have focused on the specific elements of data deletion, the field remains open to a complete solution. Motivated by these observations, we propose Unlichain as a universal blockchain architecture with unlimited data modification features for an unlimited workload. Unlichain employs a novel approach for data modification while maintaining the longest-chain rule. Furthermore, both predefined and on-demand data deletions were approved using the new indexing method.

## 4. Unlichain Architecture

**Figure 3:**
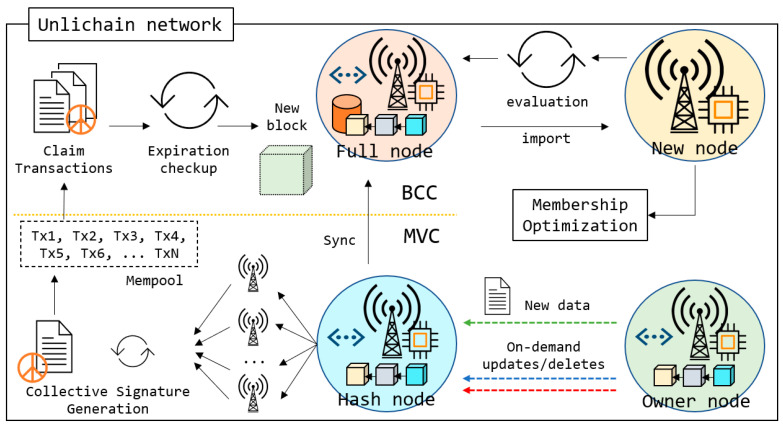
Unlichain architecture.

This section discusses the implementation and features of Unlichain architecture in detail. Because the Unlichain network is designed to handle and process big data in edge-computing environments using key-value storage [56], transactions usually contain unpredictable and different sized IoT data that may originate from sensors or media devices. The propagation of large transactions in a blockchain network is expensive in terms of storage time. To avoid long transmission delays and excessive maintenance costs, Unlichain uniquely employs two consensus procedures, as shown in Figure 3. *Meta-verification consensus* ensures fast transaction verification even prior to the creation of corresponding blocks, whereas *block-creation consensus* confirms the transactions are saved without extra delay and maintains network integrity. Furthermore, the updated membership rules allow nodes to join the network faster and participate in transaction verification. Ultimately, Unlichain employs a new data-indexing method within the blocks, which allows users to modify transactions while maintaining the trace. All these features are discussed in detail in the following subsections.

### 4.1. Nodes

As shown in Figure 3, Unlichain nodes can be categorized into four types:

- *Hash node*: The lightweight Unlichain node that stores only the chain of blocks constructed by fixed-size hashes. Hash nodes play a primary role in verifying new transactions before creating corresponding blocks.
- *Full node*: A node containing a full copy of the blockchain data. Full nodes store copies of the lightweight chain equivalent to hash blocks in addition to copies of actual data. When participating in a consensus, full nodes can verify transactions without loading the actual data by using only hashes. However, all the verified data must be further synchronized. Also, full nodes play an important role in performing deletions on the expired transactions.
- *Owner node*: Simultaneously, a node writing a new transaction becomes the owner of the data. Furthermore, when custom data modification is required, the owner node plays a primary role in the operation. Due to data privacy constraints, the on-demand data modifications are allowed only by the owner nodes. With the given privileges, the owner node has the roles in new data creation, and updates and deletes.
- *New node*: A new node is one attempting to join the network. According to the evaluation results, new nodes can be hashed or full nodes. In both cases, right after importing the hashes, the new node can participate in meta-verification consensus with the help of optimized membership rules.

Within Unlichain architecture, each node type plays a crucial role in achieving consensus. Although a hash node appears weaker in power than a full node, this node classification allows servers or computers with low computing power and limited resources to join a network. Consequently, although these nodes may not have sufficient resources to mine a block, they are crucial for achieving a meta-verification consensus and verifying newly mined blocks. Furthermore, hash node implementation allows the Unlichain network to reach a wider area, thereby improving security.

### 4.2. Consensus

The Unlichain network was designed to satisfy the data processing requirements in edge-computing environments. Because big-data-oriented systems generally encompass dynamic IoT devices, end-to-end latency is crucial for reliability. In the traditional approach, blockchain consensus focuses on heavy computation and continuous broadcasting through a network to verify transactions. However, this approach requires long transmission delays. In the context of cryptocurrencies, transaction owners can generally wait as long as they need to verify their transactions, and security has a higher priority than performance. However, the same rule cannot be applied to the IoT data. IoT devices generally involve dynamic motion that requires rapid response times. To satisfy the IoT environmental requirements while maintaining blockchain security features, Unlichain employs two types of consensus protocols.

As shown in Figure 3, Unlichain employs a meta-verification consensus (MVC) that is executed independent from block creation on each transaction creation. As discussed in Section 3.1, a traditional blockchain uses a data structure called Mempool to store pending transactions. Unlichain extended the propagation rules of Mempool data using MVC, as shown in Algorithm 1. When the owner node writes new data, a unique hash is generated by the hash nodes and broadcast to the network as a new transaction (*lines 8~11*). At this step, the hash node waits only a few confirmations enough to generate collective signature (*lines 13~18*). The number of confirmations is configurable depending on the network requirements by the global variable named *THRESHOLD*, as shown in *line 15*. After signing the transaction (*line 21*), the hash node synchronizes the corresponding data with full nodes, as shown in *line 22*. After signing, it is taken as a verified transaction and visible to the network. In the next step, the block-creation consensus (BCC) is held by full nodes and ensures that the transaction is written in the next block.
**Algorithm 1** Meta-Verification Consensus1: 2: 3: 4: 5: 6: 7: 8: 9: 10: 11: 12: 13: 14: 15: 16: 17: 18: 19: 20: 21: 22: 23: 24: 25:**Input:**  *entry* → new block to write             *network* → current state of network **Output**: *mempool* → updated list of pending transactions **Procedure**         *mempool ← network.getMempool()*        *tx ← NewTransaction(entry)*        *tx.status ← 0xFFFF…*        *tx.expire ← CURRENT_TIMESTAMP + entry.lifetime*        *Broadcast(network.getAllNodes(), tx)*         *confirmed ← new List*        *confirmations ← 0*       **while** *confirmations <= THRESHOLD* **do**               **if** *callback* **then**                     *confirmed.add(callback.ID)*                     *confirmations ← confirmations + 1*         *CS ← Hash(tx.hash + confirmed)*        *tx.sign(CS)*      * Broadcast(network.getFullNodes(), entry)*        *mempool.add(tx)*         **return** *mempool*

Traditionally, the block size has been fixed; however, the number of transactions has gradually increased. As a result, the nodes must sort transactions from Mempool to avoid exceeding the size limitations, which results in a long delay for small transactions. In the case of Unlichain, Mempool only includes metadata, which requires considerably less storage. Instead of limiting the block size, Unlichain employs a new rule that allows a node with more transactions to win the BCC. According to BCC, nodes not only need to solve the common puzzle, but also need to prove they have the most transaction in the mined blocks. Thus, miner nodes must select as many transactions as possible from Mempool and calculate the block hash. Since Mempool is visible to all nodes, claiming all pending transactions would be insufficient for winning consensus. For creating a longer list of new transactions, the nodes run an *expired data traversing procedure*, which is discussed in detail in the next sections. When verifying the mined block, other nodes check all the selected transactions with the corresponding data in the full nodes, preventing malicious nodes from adding falsified transactions to win a consensus. This simple update motivates the nodes to collect all the pending transactions in the new block. Moreover, this motivates nodes to actively verify new transactions. In simple terms, a node that verifies more transactions has a higher probability of winning a consensus.

### 4.3. Membership

Another distinguishable feature of Unlichain compared to related literature would be its optimized membership rules, as shown in Figure 3. Upon implementation of node classification, a suitable type must be selected for the new nodes. For this purpose, Unlichain employs initial evaluation-based membership rules. Whenever a new node wants to join the network, it receives the latest network metadata and performs self-evaluation. In this step, the node resources are compared with the expected block-mining difficulty and data size. If all requirements are satisfied, the node joins the network as a full node. Even if not, any type of device is allowed to join the Unlichain network as a hash node. Importing starts with Mempool, which allows the node to join the MVC directly without waiting for the full blockchain to be imported. If the node passes the evaluations, it imports the latest chain from the closest full nodes while participating in MVC. Thus, Unlichain allows nodes to participate in a suitable consensus even with limited hardware resources. Furthermore, allowing new nodes to join the consensus instantly improves network scalability.

### 4.4. Block Indexing

**Figure 4:**
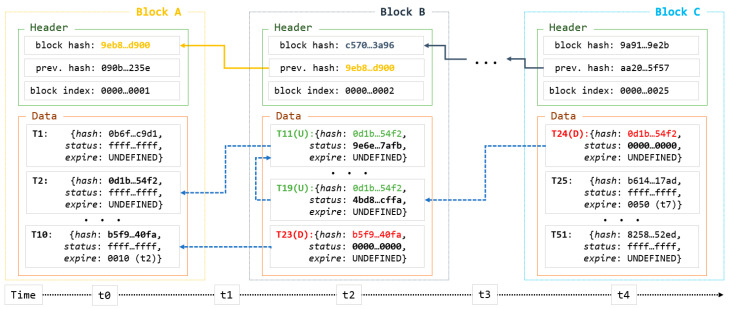
Unlichain block indexing.

Unlichain employs a new block-indexing technique to enable data update and deletion. The block structure and an example of data modification are shown in Figure 4. Methods for creating chains by linking block hashes and maintaining block data are inherited from a common blockchain structure. The figure shows only the most important components of the block structure used to implement the new method. Traditionally, the transaction *hash* and *index* are used as primary index variables. Unlichain employs a slightly modified transaction-indexing procedure with two additional variables. The hash stores a unique hash for the original data that cannot be altered. Modifications to the corresponding data are stored in the *status* variable, which defines the type of modification. 

The hash is duplicated for the modified data and the latest copy stores the latest version. Accordingly, the read method scans the hash in the reverse order of the blocks and finds the latest version. Moreover, all bits of the status field are initially set to one, indicating that the corresponding data have not yet been modified. Whenever the data are updated by an external request, a new transaction is created with the same hash as the original data, and the value of the status field is set as the hash of the updated data. The presence of a hash value in the status field indicates that the data have been updated from the previous state. In the case of deletion, the value of the status field was set to zero so that the state of the original data could be determined. Finally, Unlichain employs an *expire* field to track transaction lifetime. When the data have a predefined lifetime, the field stores the expiration time; otherwise, the value is set to UNDEFINED. Figure 4 illustrates the data-modification interactions with the corresponding records according to the block-creation timeline. The first block (*Block A*) was created at the initial point (*t0*). All records in Block A had the same value in the *status* field, indicating that the corresponding data were not modified. Before creating *Block B* in *t1*, two update requests are sent to *T2* and the expiration time is reached at *T10*. Unlichain protocol allows the creation of a new transaction for each change in the data, as opposed to deleting the old transaction. As shown in *Block B*, records *T11* and *R23* correspond to records *T2* and *T10* in *Block A*, respectively, based on the hash field. When the data are updated (*T2* → *T11*), a new hash is generated based on the updated data and is stored in the status field of the new record. However, the original data hash does not change and is instead stored in the hash field. This results in different values for the hash and status fields, indicating that the data were updated. Moreover, there may be cases where multiple updates are requested prior to the creation of a new block. For example, *Block B* includes another update for the same record (*T2* → *T11* → *T19*) and the status field stores a different value. During the delete operations (*T10* → *T23*), data are removed from the full nodes; therefore, the status field is filled with zeros. Following this rule, *T19* of *Block B* is requested to be deleted at *t3*, which means that the operation results are shown when *Block C* is created using new records. In other words, data-modification methods do not update or delete existing data or blocks from the chain. Instead, they create new transactions for modified data in the chain. This approach may waste storage when maintaining old transactions for deleted data. However, the maintained data included only metadata from the original data, which were negligible in size. Furthermore, maintaining a data-modification history enables tracing of transactions whenever needed, which in turn improves network reliability. Consequently, Unlichain achieves efficient and utilitarian data modification in the blockchain architecture. Moreover, a record of the modification history allows browsing of the data history, which provides integrity to the system.

## 5. Data Modifications

This section presents a further discussion of Unlichain architecture in terms of handling data modifications. As mentioned in previous sections, Unlichain allows for both predefined and on-demand data modifications. Based on the block-indexing example shown in Figure 3, each type is discussed in detail, and its effectiveness is compared with that of existing solutions.

### 5.1. Predefined Lifetime

Lifetime-predefined data are the basic type of data used in deletion-oriented blockchain architectures. Because the blockchain protocol is run by distributed nodes, each node must agree to a deletion to ensure its integrity. Therefore, the optimal approach writes data with a predefined expiration time, thereby enabling each node to easily verify deletions using the original data. Accordingly, Unlichain allows transactions with a predefined expiration time, which is saved in the “*expire*” field, as explained in the previous section. After verifying a transaction, the expiration time cannot be changed, and a countdown is initiated when the corresponding block is created.

Unlichain employs a new block-indexing method that enables nodes to verify deletions independently for each datum. Unit-chain consensus forces the nodes to include more data in each new block. However, according to ordinary rules, the capacity of each node is limited by its Mempool size. To obtain more transactions, nodes continuously check for older blocks and expired transactions. When an expired transaction is found, the node generates a new transaction, indicating that the old transaction has been deleted. Thus, the nodes achieve longer transaction lists and higher probability of reaching consensus. Nodes achieve this goal by running the procedure in Algorithm 2 as a part of block mining. As shown in the procedure, the entire chain is examined (*lines 7–17*) for an expired transaction. Whenever an expired transaction is found (*line 13*), it is added to Mempool (*lines 14–17*). When a new block is mined and shared with the network, the other nodes thoroughly verify each transaction in terms of expiration dates. If a malicious node attempts to delete an unexpired transaction to gain consensus, the network invalidates the request and block mining is rejected. Upon creation of a block, the full nodes perform any required deletions on the original data and update the blockchain state.
**Algorithm 2** Expired data traversing procedure1: 2: 3: 4: 5: 6: 7: 8: 9: 10: 11: 12: 13: 14: 15: 16: 17: 18: 19:**Input:**  *chain* → chain of blocks             *mempool* → new transactions to be added in a new block **Output**: *mempool* → updated list of transactions **Procedure**          **for** *i = 0; i < chain.length(); i++* **do**                 *block ← chain.get(i)*                     **for** *j = 0; j < block.data.length(); j++*
**do**                           *tx ← block.data.get(j)*                          **if**
*block.timestamp + tx.expire >= date.now()*
**then**                               *tx.status ← 0x00…00*                               *tx.expire ← NULL*                             * tx.data ← NULL*                               *mempool.add(tx)*          **return** *mempool*

This unique approach for enabling deletions among predefined transactions allows Unlichain architecture to eliminate data without deleting all the blocks from the chain. Simultaneously, it forces nodes to continuously check for expirations, thereby avoiding long delays before deletions. Moreover, the procedure requires only metadata that contain expiration information. Therefore, both meta nodes and full nodes can easily participate in the mining process.

### 5.2. Custom Modifications

Although predefined lifetime transactions are efficient in achieving deletion consensus, the IoT data environment is dynamic and cannot always provide the expected lifetime for all data. Deletion requests are typically generated dynamically under specific circumstances. Blockchain networks must handle these requests accordingly. To satisfy this requirement, Unlichain employs *delete()* and *update()* methods that enable custom data modifications. However, verification of a network is challenging when deleting data without a predefined lifetime. Consequently, these modification requests can only be approved by the owner’s device by following specified steps:

1. A deletion request is made using an IoT device. As IoT devices move unpredictably, requests are sent to the closest node.
2. The receiving node verifies the request source by using the original data. Because only the data owner can make modifications, a request from a different IoT device is rejected by the node.
3. The receiving node then broadcasts the request to the network. In this step, the delete() or update() API is used, depending on the request type.
4. Network nodes verify the source once more and confirm the transaction, thereby enabling modifications.
5. The new transaction indicating the data modification is added to the Mempool.

When a corresponding block is created, all nodes update the blockchain state based on new transactions. Thus, Unlichain achieved an efficient approach to custom data modification.

The final step of data deletion is synchronizing the deleted transactions with the full nodes. Full nodes execute the data removal procedure during every new block creation, as shown in Algorithm 3. The procedure takes the new block as input parameters. Since predefined and custom deletions are saved as new transactions in the global blockchain state, the new block keeps the information about both deletions. First, the entire block is checked for transactions that contain the deletion information, as shown in *lines 5–10*. The transaction is deleted if the *status* field is set to *0x00…00* (*line 9*). When the deleted transaction is found, the address of its corresponding data is deleted (*line 10*). It is a simple procedure that does not have much energy in full nodes. However, executing the same steps in each block creation allows all full nodes to keep updating the latest state of the blockchain without broadcasting and confirmations.
**Algorithm 3** Data removal procedure1: 2: 3: 4: 5: 6: 7: 8: 9: 10: 11:**Input:**  *block* → new verified block **Procedure**        **for** *i = 0; i < block.data.length(); i++)* **do**             *tx ← block.data.get(j)*               **if**
*tx.status == 0x00…00*
**then**                   *tx_old ← getTransaction(tx.hash)*                   *Delete(&tx_old.data)*

### 5.3. Efficiency

Because data deletion is a central advantage of Unlichain, it is important to examine its efficiency. As discussed in previous sections, Unlichain employs an efficient consensus technique to verify data modifications, allowing for both deletions and updates when custom requests are sent by the owner’s device. When these modifications are made, the result is reflected in the subsequent block and the state is updated for all nodes. Ultimately, it is safe to say that Unlichain achieves almost constant delay when deleting data. The deletion efficiency of Unlichain has also been proven using a computational approach. First, let us consider the general case of performing deletions on the distributed network. According to the blockchain hashing protocol, the deletions can only be performed in the opposite order of block creation. Algorithm 4 shows the calculation steps of possible delay caused by this rule. When we want to calculate the expected delay, the first step is to find the optimal case without any block dependency. In this way, the deletion only waits until the next block generation, in other words, only one block generation interval (*lines 7~10*). The next step is calculating the dependent blocks until the data become available to delete. The same interval delay and garbage collection delay (*GC_DELAY*) are added for each dependent block (*lines 12~17*). For comparison purposes, let us generalize the procedure for all deletion protocols. The block creation interval (*line 9*) can be taken as the unavoidable constant delay. Also, additional intervals for each dependent block (*line 14*) may vary for different blockchains. So, it can be taken as a configurable delay function. Finally, the garbage collection delay (*line 15*) may not necessarily take a role in creating delays since it can be performed in the background. Instead, we can introduce a control function for extra delay for block indexing. Based on these observations, and motivated by adapting the optimal control law [57] to the deletion delay cost scenario, the mathematical form of the generalized delay function would be as follows:

(1) $$D=C_{0}+\sum_{k=1}^{n}\left(G\left(k\right)+u\left(k\right)\right),\;$$

where *C*_0_ is a constant for deleting the selected data or blocks. Assuming that there are n parent or child blocks on which the selected data are dependent, *G*(*k*) is defined as the delay function for notifying or deleting each parent/child block, and *u*(*k*) is the control function for directing between blocks.
**Algorithm 4** Generalized delay calculation procedure1: 2: 3: 4: 5: 6: 7: 8: 9: 10: 11: 12: 13: 14: 15: 16: 17: 18: 19:**Input:**   *tx* → transaction to be deleted               *chain* → current state of blockchain **Output**:  *D* → expected delay until the deletion **Procedure**         *last_block ← chain.get(chain.length - 1)*        *prev_block ← chain.get(chain.length - 2)*        *interval ← last_block.timestamp – prev_block.timestamp*        *D ← interval*         *tx_block ← chain.get(tx.block)*        **while** *tx_block != last_block* **do**              *D ← D + interval*              *D ← D + GC_DELAY*               *tx_block ← chain.get(tx_block + 1)*        **return** *D*

As discussed in Section 3, LiTichain focuses on deleting the last block. If data from the middle block were selected for deletion, there would be a delay until all child blocks were located after the selected block was deleted. The deletion cost and delay for each child block are equivalent and defined as *G*(*1*) *= G*(*2*) *=… = G*(*n*). Furthermore, there is no requirement for a control or preprocessing function prior to each block deletion, because the blocks are located in order. Therefore, *u*(*0*) *= u*(*1*) *= … = u*(*n*) *= 0*. Based on these observations, the total delay function for LiTichain (D_L_) for deleting arbitrarily selected data from LiTichain can be defined as

(2) $$D_{L}=C_{0}+n\times G\left(n\right),$$

The equation shows that the effect of *u*(*k*) is zero, where LiTichain does not need to depend on routing between blocks to perform deletion. However, deleting each of the last blocks marked for deletion is expensive. Therefore, the deletion cost is defined as *n ×G*(*n*).

The SDM technique focuses on forgetting the first n blocks by mining a new summary block and changing its location within the genesis block. In addition to LiTichain, SDM does not delete each block individually; instead, it creates a single new block. Thus, $\sum_{k=1}^{n}G\left(k\right)\;=G(0)$. Moreover, the control function costs for each forgotten block are equivalent until the summary block is created. Thus, *u*(*0*) *= u*(*1*) *= … = u*(*n*). Therefore, the final equation for the deletion delay in the SDM (*D_SDM_*) of arbitrarily selected data is

(3) $$D_{SDM}=C_{0}+G\left(0\right)+n\times u\left(n\right),\;$$

In other words, deletion is performed by creating only one block using SDM; thus, the cost is *G*(*0*). However, before creating the blocks, the SDM reads all the deleted blocks to create summary transactions, and there is an additional cost *n ×u*(*n*).

Finally, unlike the other two methods, Unlichain does not rely on parent or child blocks to delete the data. Instead, the selected data can be deleted from the full nodes, irrespective of the reference hash location in the blockchain. Thus, *G*(*1*) *= G*(*2*) *=… = G*(*n*) *= 0*, and *u*(*0*) *= u*(*1*) *= … = u*(*n*) *= 0*. Substituting these values into the original equation yields the following delay for Unlichain (*D_U_*):

(4) $$D_{U}=C_{0},$$

Equations (2) and (3) are still ambiguous in drawing a conclusion regarding better performance, as they rely on configuration-dependent variables, such as *G* and *u*. However, Equation (4) proves that Unlichain deletion offers superior performance with a nearly constant time delay.

## 6. Discussion

This section discusses the importance of data modifications in blockchain architecture and Unlichain’s advantages over the existing state-of-the-art solutions. 

Table 2 shows the key differences among four blockchain solutions employing data deletions in different consensus techniques. LiTichain uses a permissioned network to achieve more secure and faster transaction confirmation. The authors mention that PBFT can be an acceptable consensus technique considering the organization-managed network environment. Within this scope, they employ arrival-time and expiration-time ordering techniques that locate the blocks in the tree-like structure based on their expiration time. When the expiration time is reached, the block can be deleted if it does not have a child block. Using this approach, the write performance can vary depending on the configuration and applied environment. In the best-case situations, PBFT consensus may achieve a promisingly fast performance in private networks. Hillman et al. introduced the concept of selective deletions (SDM) free of specific consensus dependency. The authors claim that the concept can be applied to any consensus. The deletion approach involves creating the summary blocks in certain intervals that tend to forget expired data from previous blocks. The write performance is highly dependent on the applied consensus technique, and it can either be very fast (private network) or slow (PoW). The deletion performance, conversely, depends on the creation of summary blocks and the block creation interval. Hyperledger Fabric also employs a configurable consensus based on the organization’s needs. However, in most cases, the transactions are confirmed using the endorsement technique instead of common consensus. Also, this benefits in achieving very fast write performance (~2000 TPS). Hyperledger Fabric also offers a deletion technique by data pruning. This approach stores the actual data in world-state databases as key-value pairs. The corresponding hash is shared in the blockchain. When the data state is changed to delete, the key-value pairs can be deleted from the world state. However, data pruning is not automated but depends on the administrator command. So, there might be unpredictable delays before the data are deleted.

On the other hand, Unlichain employs the combination of MVC and BCC for verifying and confirming transactions. First, MVC plays an essential role in achieving scalability in write performance. As Algorithm 1 states, the MVC waits for the confirmations only long enough to sign the transaction. As the network becomes larger, more confirmations are possible. Naturally, it results in increasing the write performance. Based on the evaluations for the simulation environment, which will be discussed in the next section, Unlichain can achieve over 1000 TPS, and this performance increases proportional to the network size. Considering the public blockchain scenario, this performance is very promising in big-data processing. In addition to MVC, Unlichain employs node classification by separating full and meta nodes. Apart from Hyperledger Fabric, it creates the opportunity to automate data cleaning for each block creation. Considering these advantages, Unlichain achieves instant data deletion.

## 7. Evaluations

Evaluations of Unlichain performance and its comparison with other techniques were performed in two ways: (1) in an edge-computing environment as the target goal and (2) MATLAB (version: R2019a) simulations on different use cases.

### 7.1. Environment and Workload Setup

The evaluations were performed on a sample edge-computing environment setup using different types of nodes, as listed in Table 3. Because Unlichain architecture is designed for both high- and low-computing nodes to achieve mutual consensus, it is important for the testing environment to satisfy these requirements. For this purpose, Amazon EC2 instances and local servers were selected as examples of higher resources. Moreover, embedded devices, such as Jetson AGX Xavier, were selected to fill the network with fewer computing nodes. All of these nodes are connected to each other as an edge-computing network. Thus, the Amazon EC2 instance is not used for cloud storage but instead as an ordinary edge-node simulating different locations in the network. Thus, the simulated environment helps to evaluate the locality factors in data transmission. Moreover, considering the computing capabilities of the embedded devices, the hardware resources of the three Jetson AGX Xavier boards were orchestrated for one edge node (one master and two workers). This technique allows a blockchain node to remain active, even if the number of tasks increases at the edge. The master node ensures that one board always runs an unlicensed instance. Considering the big-data environment, the storage parameters shown in Table 3 may seem limited. However, the primary purpose of the evaluations is to address the data deletion capabilities of the proposed blockchain. So, the workloads are designed in a deletion-intensive manner where the total storage usage would stay the same over 500 GB. Even in the worst cases, the total storage usage would increase to 2.5 TB. So, the nodes are capable of dynamically allocating enough storage in runtime.

**Figure 5:**
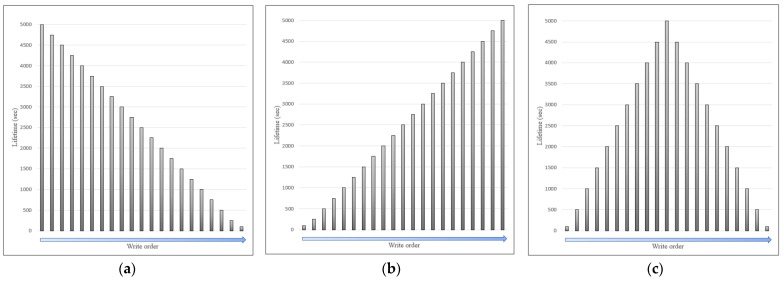
Evaluation workload setup: (**a**) Workload A; (**b**) Workload B; (**c**) Workload C.

Furthermore, three evaluation workloads were prepared, as shown in Figure 5. Because the existing approaches cannot perform on-demand deletions, the default configuration of workloads encompasses only predefined lifetime data. A total of 2.5 million entries were uniformly distributed for more than 4900 different lifetimes, ranging between 100 and 5000 s. In this scenario, each range includes approximately 500 entries. According to the nature of the blockchain, all data are converted to bytecodes before storage is saved. Moreover, the Unlichain protocol uses metadata based on a fixed-size hash. This implies that the size of the data does not affect performance. Based on these observations, entries were generated using a random algorithm with sizes varying from 50 B to 1 MB to simulate an edge-computing environment. All the data were rearranged into three types of workloads, as shown in Figure 5. The workloads were designed as the longest first in Figure 5a, the shortest first in Figure 5b, and bimodal distributions in Figure 5c. For an independent evaluation, each workload was mixed with a certain percentage of undefined lifetime data. For example, Workload A (30%) indicates that Workload A was employed, and 30% of the entries had no predefined lifetimes.

### 7.2. Delete Performance

**Figure 6:**
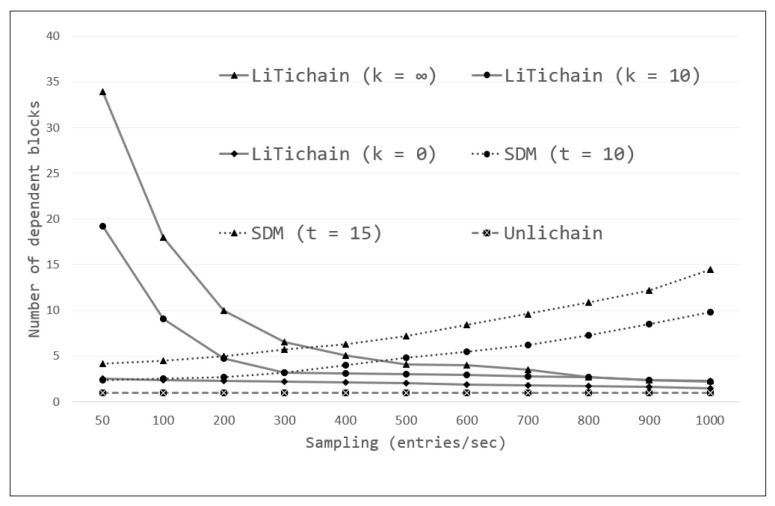
Delete performance.

For real-world evaluations, we selected prototypes of LiTichain and SDM blockchains as the sample LBD and FBD methods, respectively. Because none of these methods have been deployed in public blockchains, we used the previously described simulation edge-computing environment. To ensure a realistic and fair evaluation, the written order was distributed randomly without a specific order for a random workload. The block dependencies for the workloads with three blockchains are plotted in Figure 6. The evaluations confirm the observations and mathematical analysis in Section 5.3. As described in Section 3.2, LiTichain implemented a k-height insertion architecture with a configurable variable k; smaller values for k result in following only the expiration time ordering, where the linear order of blocks is lost, in turn providing more space to locate the latest blocks and minimize deletion delay. Three values of k (∞, 10, and 0) were used for the evaluations. As shown in the figure, by decreasing the value of k, LiTichain achieves more stable performance, changing from an exponential rate to an almost constant performance. This behavior can also be explained using Equation (2), which shows the block dependency of LiTichain. However, setting k = 0 makes the LiTichain architecture the most vulnerable to possible attacks; thus, its performance advantage is leveraged. Furthermore, as the sampling size increased, LiTichain still converged after approximately two block dependencies on average, which is unacceptable in an edge-computing environment. In contrast, SDM exhibited a more unpredictable performance than the FBD method as its deletion performance depends mostly on the control function, as shown in Equation (3). In the real-life environment, the cost of control function increases as the data size becomes larger. According to the SDM protocol, summary blocks were generated based on a threshold. Consequently, the number of block dependencies approaches a threshold value with an increase in sampling. Under sample threshold values of 10 and 15, the figure shows a linear increase in SDM dependencies. Because Unlichain does not rely on deleting entire blocks to perform data deletion, it exhibits the most promising results with a constant performance in the edge environment. The unit-chain protocol motivates the confirmation of data modification in each subsequent block, keeping the dependent block constant at one.

### 7.3. Block Height

**Figure 7:**
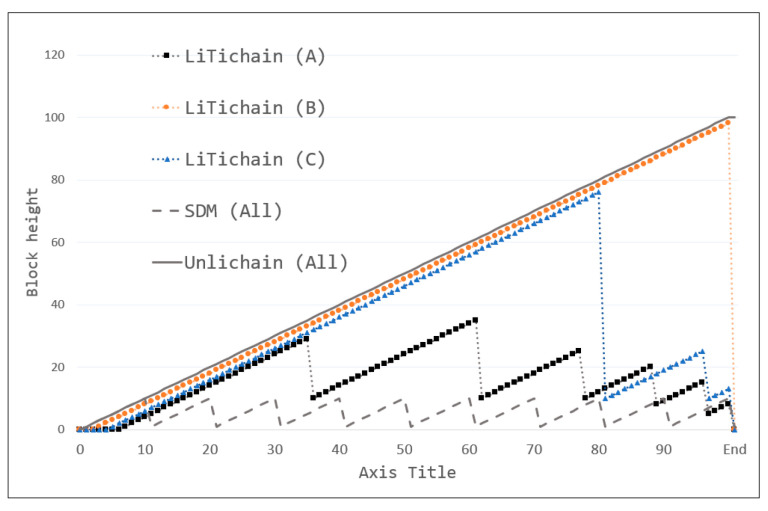
Block height.

The block height is a fundamental blockchain feature that maintains security. Because new blocks are created owing to certain difficulties, gaining height requires multiple time-consuming iterations for the same procedure. Even if malicious nodes provide sufficient resources to recalculate hashes, they may quickly reach the original block height owing to time constraints. Consequently, maintaining the latest block height and following the longest-chain rule is a basic, unreplaceable blockchain protocol. The evaluation of the maintenance of block height is also an important factor. The results of this evaluation criterion are shown in Figure 7, with the default configurations used for all methods. The block height for LiTichain varies with respect to the workload according to its architecture, whereas SDM and Unlichain maintain the same block height for all workloads. As shown in the results, LiTichain yielded the best performance on Workload B. However, this also proves that LiTichain exhibited the worst performance on the exact workload in terms of delete latency. Furthermore, changes in block height are unpredictable for Workloads A and C because certain blocks rely on the next blocks before deletion. Once the requirements were satisfied, several blocks were deleted simultaneously, thereby reducing the chain height. It is also noteworthy that the LiTichain block height converges to zero at the end of the workload, indicating that the architecture does not have a chain in finite-lifetime environments. In contrast, SDM repeats the block height based on the summary block interval, with new blocks created until the interval is met and replaced by one block. Consequently, SDM has only one block at the end of the workload. In terms of block height, Unlichain exhibited the most stable performance, maintaining all blocks irrespective of the deleted data. Even for deleted data, Unlichain maintains the metadata in the blockchain. Thus, only Unlichain maintains the longest-chain rule and achieves sufficient reliability.

### 7.4. Storage Efficiency

**Figure 8:**
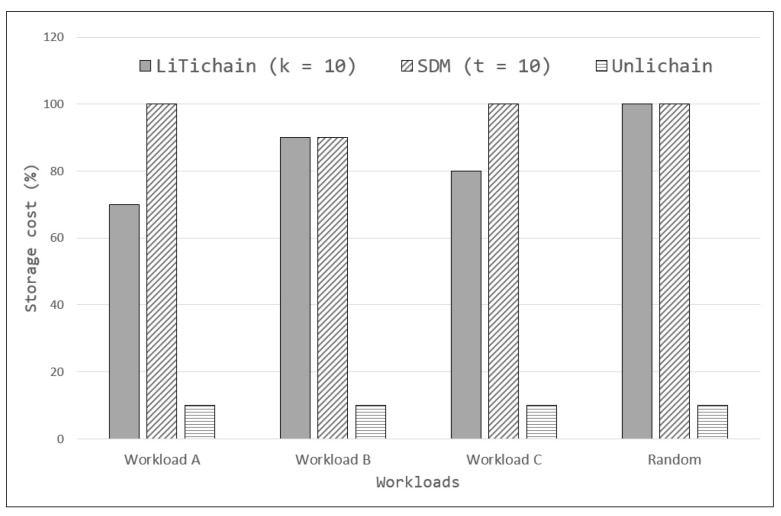
Storage efficiency.

Subsequent evaluations of the three blockchains were performed to determine the storage efficiency while employing the deletion technique. The storage cost was recorded for each blockchain after each workload was tested; the results are shown in Figure 8. In these evaluations, the storage cost defines the storage usage from data-lifetime expiration to when the next value is written. This indicates that the storage is used even when it is freed for the next data to be written. With the help of higher values for the configurable variable k, LiTichain achieved better performance than SDM with Workloads A and C; however, it still could not handle random workloads. Generally, both LiTichain and SDM use at least 70% of the storage and incur unacceptable costs in terms of efficiency. By contrast, Unlichain deletes the data within the next block creation and preserves the corresponding metadata in the blockchain. The total cost to maintain the data until synchronization was complete and the metadata size reached was up to 10%, relative to the actual size. Although this may seem unnecessary for storage usage, it is more efficient than the other two methods while offering additional reliability features.

### 7.5. MATLAB Simulations

**Figure 9:**
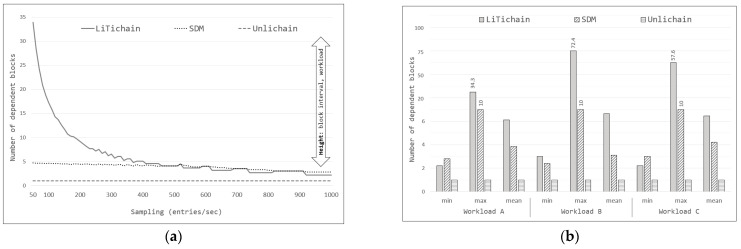
MATLAB simulation results: (**a**) overall; (**b**) block dependency.

For comparison, prototypes of LiTichain, SDM, and Unlichain were implemented in the MATLAB environment, and the evaluation results are shown in Figure 9. To obtain results, each method was tested for each workload at different sampling intervals. In these experiments, sampling defined the number of entries per second. Assuming that the blocks were created in order at constant time intervals, the average result for each interval was recorded. The simulations showed that the general behavior of all methods came to a general view for all workloads, as depicted in Figure 9a. The figure shows the average number of dependent blocks that the expired data must wait for. Because the LiTichain and SDM methods focus on deleting entire blocks, the expiration times of the first-to-expire data from each block were used for evaluation. LiTichain exhibited the worst overall performance for smaller samplings, with an exponential decrease as the sampling increased. This may be explained by reliance on subsequent blocks to expire before deletion. Because the blocks are generated in order with a specific time interval, the lifetime count starts only after the data are written to each block. As the definition states, new blocks are always be created prior to the expiration time for the previous block, which means that in small samplings, the first blocks have to wait until the last block is deleted, resulting in an excessively high dependency. Even for different workloads, the general scenario is comparable. Unlike LiTichain, the SDM method does not rely on the lifetime of the next block, allowing blocks to be deleted immediately upon expiration. Consequently, SDM exhibited more promising results, with a linear decrease as the sampling increased. Even though the general view is similar, the heights of the graphs changed according to different workloads and block-creation intervals. From the simulations, Unlichain produced the most promising results with an almost constant dependency for all workloads and cases. Unlichain protocol does not require block deletions to perform the operation; instead, it relies solely on the validation of new transactions. As a result, there is always a single-block dependency: a new block to write the deletion record.

Figure 9a provides an overall view of the scenario, whereas Figure 9b presents more detailed information pertaining to the simulation results, showing the minimum, maximum, and mean values of the block dependencies for all the workloads. As shown in the figure, the maximum number of block dependencies for Workloads A, B, and C were approximately 34, 73, and 58, respectively. Furthermore, the minimum (2–3) and mean (6–10) values differed according to workload. A similar conclusion can be drawn from the SDM results for the minimum and mean cases (between 2 and 5). However, the maximum block dependency for the SDM was always 10 for all workloads according to its configuration. These observations prove that, although the height of the graph changes according to the workload, the overall trend remains the same. However, Unlichain achieved a constant block dependency, as observed in all other evaluations.

These evaluations prove that Unlichain architecture outperforms existing approaches in all cases in terms of performance and reliability.

## 8. Conclusions

The integration of blockchain into an edge-computing environment presents a new research gap related to data deletion. Even though there are existing solutions with data deletion possibilities in blockchain, they all suffer from long delays not practical in big-data systems. This study proposes a novel Unlichain approach to enable data-modification features in blockchain architecture. Unlichain employs a meta-verification consensus for achieving instant verifications of transaction metadata. Also, the updated block creation consensus allows all pending transactions to be written in the next block without further delays. Moreover, the new block indexing technique makes the data modification operations possible for predefined lifetime data and on-demand delete requests. The block indexing technique allows Unlichain to maintain the chain height even after data deletions following the traditional longest-chain rule. Additionally, the meta and full node classification allows participation in the Unlichain consensus even with limited hardware resources. Extensive evaluations were performed in both edge-computing and MATLAB environments. According to the results, Unlichain maintains stable performance for data deletion with negligible delay. Compared to block dependencies of existing solutions, Unlichain achieves instant verification on deletion and update requests. Moreover, Unlichain has proven to be the most reliable in data modification and storage efficiency and in maintaining basic blockchain protocols.

## Figures and Tables

**Table 1:** Comparison of related literature.

Ref	Type	D ^1^	U ^2^	OC ^3^	LCR ^4^	NC ^5^	IC ^6^	SD ^7^	MO ^8^
Bitcoin [1]	Public	X	X	X	✓	X	X	X	X
Ethereum [2]	Public	X	✓	X	✓	✓	X	X	X
LiTichain [11]	Permissioned	✓	X	✓	X	X	✓	X	X
Hillman et al. [12]	Public	✓	✓	✓	X	X	X	✓	X
Kuperberg et al. [13]	Permissioned	✓	✓	✓	X	✓	X	✓	X
Hyperledger Fabric [18]	Permissioned	✓	✓	X	X	✓	X	✓	X
Sayeed et al. [19]	Permissioned	✓	✓	X	X	✓	X	✓	X
Kanboubi et al. [26]	Private	✓	✓	X	✓	X	✓	✓	X
Guo et al. [31]	Permissioned	✓	✓	X	X	✓	✓	✓	X
IOTA [41]	Public	X	X	✓	✓	✓	✓	X	X
Unlichain (Proposed)	Public	✓	✓	✓	✓	✓	✓	✓	✓

^1^ Delete, ^2^ Update, ^3^ On-Chain data handling, ^4^ Longest-Chain Rule preservation, ^5^ Node Classification, ^6^ Instant Confirmation, ^7^ Selective Deletion, ^8^ Membership Optimization. ✓: feature is available; X: feature is not available.

**Table 2:** Key differences among four blockchain solutions.

Ref.	Consensus	Deletion Method	Write Performance	Delete Performance
LiTichain [11]	Permissioned/ PBFT	Sort the blocks in deletion order: First to delete is confirmed last	Configuration dependent (private/fast)	Configurable (instant at k = 0)
Hillman et al. [12]	Public/ Configurable	Creating a summary block: Forget the deleted data	Consensus dependent	Configurable: BI ^1^ × SBI ^2^
Hyperledger Fabric [18]	Permissioned/ Endorsement	On-demand data pruning on world state: Transaction history remains in blockchain	Configuration dependent (very fast ~2000 TPS ^3^)	Dependent on external command
Unlichain (Proposed)	Public MVC/BCC	Automated data cleaning on each block creation: Transaction history remains in blockchain	Proportional to network size (1000 TPS~)	Instant

^1^ block creation interval, ^2^ summary block creation interval, ^3^ transactions per second.

**Table 3:** Node specifications.

Name	CPU	DRAM	Storage
Jetson AGX Xavier	4 × ARMv7 Processors	16 GB	1 TB
Local servers	8 × AMD Ryzen 7 1700 CPUs 3.0 GHz	16 GB	3 TB
	2 × Intel Core i5 CPUs 3.3 GHz	8 GB	3 TB
Amazon EC2 (i3en.xlarge)	4 × vCPUs 2.5 GHz	32 GB	2.5 TB

## Data Availability

Not available.
